# Breastfeeding: Benefits to Infant and Mother

**DOI:** 10.3390/nu16193251

**Published:** 2024-09-26

**Authors:** Robert Roghair

**Affiliations:** Stead Family Department of Pediatrics, Carver College of Medicine, University of Iowa, Iowa City, IA 52242, USA; robert-roghair@uiowa.edu

## 1. Introduction

The provision of human milk to newborn infants is one of the most effective ways to reduce infant mortality. It has been estimated that nearly one million children’s lives could be saved every year if all children were optimally breastfed [1]. Later in life, breastfed children perform better on intelligence tests, are less likely to be overweight or obese and less prone to diabetes [2]. Additional benefits are seen for birthing parents that provide their own milk for their infants, including a reduction in the rates of many types of cancer and decreased metabolic or cardiovascular disease [3,4]. Based on this evidence, the World Health Organization (WHO) and the United Nations International Children’s Emergency Fund (UNICEF) recommend that children initiate breastfeeding within the first hour of birth and be exclusively breastfed for 6 months [5]. Unfortunately, more often than not, infants receive non-human milk feedings before 6 months, and the provision of breast milk often ends before the evidence-based target of at least 2 years. Beyond the global imperative, disparities in breastfeeding rates within countries are striking and the variable rate of breastfeeding across racial or ethnic groups and among individuals with different socioeconomic statuses is an under-appreciated public health crisis.

This Special Issue “Breastfeeding: Benefits to Infant and Mother” was created to develop evidence-based recommendations to increase the initiation and maintenance of breastfeeding worldwide and further expand upon the health benefits of human milk across diverse populations. This effort was successful with dozens of submissions subjected to rigorous peer review, resulting in twelve outstanding contributions to the medical literature in this important area. This editorial is intended to assist in the dissemination of this newfound knowledge and awareness. In turn, it is hoped that caregivers and care providers will be able to use this information to increase breastfeeding rates with positive impacts on child and adult health.

## 2. Overview of Published Articles

In the first contribution, Elgzar et al. utilized a retrospective, cross-sectional study to examine the correlation between specific components of maternal ideation on exclusive breastfeeding (EBF) practices in Saudi Arabia (contribution 1). The reported rate of EBF at 6 months within this cohort (41%) is consistent with global rates, supporting the widespread applicability of the inquiries. The investigation found that each construct of maternal ideation, including cognitive aspects (adequate knowledge and positive beliefs), psychological dimensions (self-efficacy) and social dimensions (social influence, descriptive and injective norms) were positive predictors of EBF, as were traditional factors, including maternal age, mode of delivery, occupation, and education.

Olcina Simón et al. added another layer to the investigations of Elgzar et al. by delving into the role of cognitive and social dimensions in the persistence of colostrum avoidance among a majority of women in rural Ethiopia (contribution 2). In their cross-sectional study, only 3% of birthing parents started breastfeeding within an hour of birth and 56% practiced colostrum avoidance. Maternal educational level, living conditions, beliefs about the dangers of colostrum, and attendance at delivery by relatives rather than healthcare providers predicted colostrum avoidance. Unfortunately, inaccessible health care contributed to limited or no prenatal care for most of the study participants, and this likely contributed to the persistence of the traditional feeding practices despite their association with increased infant mortality.

D’Adamo et al. published a related time-course investigation into the impact time since delivery on the breast milk content of presepsin, a truncated, soluble form of CD14 that has been used as a short-term biomarker for infection (contribution 3). Their prospective study demonstrated reductions in presepsin with advancing gestational age at delivery and elapsed time since delivery with relatively minor variation seen based on mode of delivery or infant gestation. Notably, presepsin levels are markedly higher in colostrum than transitional or mature milk, especially among preterm infants. While the impact of breast milk presepsin on gut health and innate immunity is an area of ongoing investigation, these data support the importance of colostrum feedings, especially in the context of preterm delivery.

Although it is certainly not available to all families, donor human milk can be used as a bridge towards the provision of maternal milk or as a substitute for maternal milk during prolonged neonatal hospitalizations. In those situations, it is important for donor milk to replicate the characteristics of maternal milk, and in this regard, the article by Vass et al. examined the impact of demographic parameters on the fatty acid composition of donor milk (contribution 4). While Holder pasteurization significantly reduced the content of multiple medium and long chain fatty acids, there were significantly increased levels of other fatty acids. Similar changes were seen, independent of pasteurization, based on mode of delivery with smaller effects identified in relation to the gender of the donor’s infant. While the absolute differences in fatty acid content were relatively small, it is clear that further research is needed to define the effects of various pasteurization methods and donor selection strategies on the composition of donated human milk.

Returning to the investigation of the correlates of early EBF success, Verea-Nuñez et al. performed a cross-sectional study at a certified baby-friendly hospital in Spain with an EBF rate during admission of 73% (contribution 5). While there was an overall positive attitude towards breastfeeding, this was further enhanced by avoidance of pacifiers and the provision of proper breastfeeding information from healthcare providers. Beyond having a positive attitude, having a positive prior breastfeeding experience and an infant that did not require specialized care was associated with increased likelihood of EBF throughout the newborn admission. These results emphasize the importance of assisting initial breastfeeding efforts for benefits that extend beyond the current dyad to subsequent births.

A second cross-sectional contribution from Saudi Arabia by Al-Thubaity et al. focused on the determinants of maternal self-efficacy in the ability to breastfeed satisfactorily, a key component of EBF ideation, especially when extra effort or persistence are needed in the context of unexpected barriers or challenges (contribution 6). Reinforcing the findings of Elgzar et al. and Verea-Nuñez et al., maternal self-efficacy over the first 6 months following delivery was significantly influenced by a positive maternal attitude, as well as maternal occupation, education, and past EBF experiences. It is worth emphasizing the study’s important, but perhaps not surprising, finding that housewives were nearly twice as likely to have a high EBF self-efficacy compared with working mothers, highlighting a need for improved workplace and parental leave policies.

The prospective cohort study performed in southern Brazil by Bizon et al. further emphasized the importance of a positive maternal attitude in the maintenance of EBF by directly correlating the level of maternal satisfaction at 1 month with ongoing EBF (contribution 7). In a multivariate model that corrected for maternal age, maternal education, cohabitation, and early breastfeeding difficulties, mothers with a higher level of satisfaction at 1 month were over four times as likely to be EBF at 6 months. While the results emphasize the importance of early interventions to achieve maternal satisfaction, the EBF rate at 6 months of only 24% among the cohort with high levels of early satisfaction emphasizes the need for additional and ongoing assessments and interventions.

In a complimentary investigation, Vila-Candel and colleagues explored the importance of health literacy in the prevention of breastfeeding abandonment (contribution 8). Their multicenter study across four regions of Spain prospectively assessed breastfeeding attrition over 6 months with rates gradually declining from 82% to 43%, consistent with global rates of breastfeeding abandonment. Health literacy, along with mobilization during labor and spontaneous labor were significantly associated with a reduced likelihood of breastfeeding abandonment, perhaps a reflection of improved feelings of maternal self-efficacy or control over the perinatal events. Interestingly, unlike the results of other studies within this Special Issue, maternal education level or economic status did not predict either health literacy or breastfeeding discontinuation, emphasizing the need for region-specific investigations.

With multiple studies demonstrating the importance of maternal support or education in the development of maternal self-efficacy, Rodríguez-Gallego led a remarkable randomized clinical trial that tested the effectiveness of a monthly midwife-led postpartum support group on breastfeeding outcomes at 4 months among 382 women from Andalusia (contribution 9). At 6 months, the rate of EBF was significantly higher in the intervention arm, and this appeared to be mediated by an increase in perceived self-efficacy. In a second contribution from the same interventional study, women in the postpartum support group also had significantly reduced Edinburgh Postnatal Depression Scale scores at 4 months (contribution 10). At both 2 months and 4 months, higher depression scores were significantly associated with reduced EBF rates. Beyond the correlation with EBF rates, the mitigation of maternal depression through enhanced support group participation has implications for the long-term health of both mother and child.

Continuing the shift towards long-term outcomes, Melov et al. investigated the association of prior breastfeeding with subsequent maternal health (contribution 11). In their retrospective cohort, an impressive 62% of multiparous women in Sydney, Australia, breastfed over 6 months in their prior pregnancy, although disparities were noted in association with maternal age, ethnicity, and economic status. Overall, high intensity breastfeeding for at least 3 months in the prior pregnancy was associated with nearly a 50% reduction in the odds of an abnormal fasting glucose in the subsequent pregnancy and a tendency towards a reduced likelihood of being diagnosed with gestational diabetes.

Finally, the long-term outcomes of breastfed infants were evaluated by Libuda and colleagues (contribution 12). Their observational study allayed concerns that breastfeeding could prevent early childhood eczema in higher-risk infants at the cost of a rebound increase in allergic diseases through young adulthood. Overall, beyond the known protection from early eczema, there was no association between breastfeeding and healthcare provider-diagnosed allergic diseases. There was, however, an interesting bidirectional interaction, based on the presence or absence of early eczema, between breastfeeding and long-term allergic rhinitis risk in those with no family history of allergic diseases.

## 3. Conclusions

This compilation of articles has significantly expanded the depth of our understanding of the factors that facilitate breastfeeding initiation and continuation, as well as the public health implications of those efforts. The role of maternal ideation, in particular self-efficacy, assumed a prominent role in this Special Issue, and for good reason, as multiple publications demonstrate the importance of maternal satisfaction in the decision to continue breastfeeding with implications for the health of the infant, the mother, and potential future pregnancies. Given the ongoing need to increase breastfeeding rates, it is encouraging that many of the factors that contribute to a positive maternal attitude can be modifiable.

Only when healthcare providers screen for and identify suboptimal levels of maternal knowledge or self-efficacy can discussions begin with families regarding factors that might be contributing. Those conversations can, in turn, open the door to possible interventions to promote a more positive experience for the mother–infant dyad and the prevention of premature breastfeeding abandonment. As the contributions in this Special Issue attest, there are important roles in this process for all members of the multidisciplinary healthcare team, including lactation consultants, midwives, and additional community-based allies that contribute towards our shared understanding of the benefits of human milk.

Beyond encouragement to implement expanded screenings and evidence-supported quality improvement projects, the contributions gathered for this Special Issue identify important areas for future research. These areas include the optimal design of breastfeeding education programs and/or interventions in developing as well as developed countries. While the public health importance of breastfeeding is a clear call for an overall increase in resource allocation by policy makers, in particular an improvement in parental leave policies and universal screenings, and because resources remain limited, there is a need to identify ways to risk-stratify individuals for need-based and targeted educational interventions to achieve both effective and sustainable effects in all communities.
